# Effects of elevated atmospheric CO_2_ concentrations, clipping regimen and differential day/night atmospheric warming on tissue nitrogen concentrations of a perennial pasture grass

**DOI:** 10.1093/aobpla/plv094

**Published:** 2015-08-13

**Authors:** Astrid Volder, Roger M. Gifford, John R. Evans

**Affiliations:** 1 Department of Plant Sciences, University of California – Davis, Davis, CA, USA; 2 CSIRO Agriculture, Canberra, Australian Capital Territory 2601, Australia; 3 Division of Plant Sciences, Research School of Biology, The Australian National University, Linnaeus Building 134, Canberra, Australian Capital Territory 0200, Australia

**Keywords:** C : N ratio, climate change, defoliation, grassland, tissue quality

## Abstract

Forecasting the effects of climate change on nitrogen (N) cycling in pastures requires an understanding of changes in tissue N. We examined the effects of elevated atmospheric CO_2_ concentration, atmospheric warming and simulated grazing (clipping frequency) on aboveground and belowground tissue N concentrations and C : N ratios of a C3 pasture grass. *Phalaris aquatica* L. cv. ‘Holdfast’ was grown in the field in six transparent temperature gradient tunnels (18 × 1.5 × 1.5 m each), three at ambient atmospheric CO_2_ and three at 759 p.p.m. CO_2_. Within each tunnel, there were three air temperature treatments: ambient control, +2.2/+4.0 °C above ambient day/night warming and +3.0 °C continuous warming. A frequent and an infrequent clipping treatment were applied to each warming × CO_2_ combination. Green leaf N concentrations were decreased by elevated CO_2_ and increased by more frequent clipping. Both warming treatments increased leaf N concentrations under ambient CO_2_ concentrations, but did not significantly alter leaf N concentrations under elevated CO_2_ concentrations. Nitrogen resorption from leaves was decreased under elevated CO_2_ conditions as well as by more frequent clipping. Fine root N concentrations decreased strongly with increasing soil depth and were further decreased at the 10–60 cm soil depths by elevated CO_2_ concentrations. The interaction between the CO_2_ and warming treatments showed that leaf N concentration was affected in a non-additive manner. Changes in leaf C : N ratios were driven by changes in N concentration. Overall, the effects of CO_2_, warming and clipping treatments on aboveground tissue N concentrations were much greater than on belowground tissue.

## Abstract (precis)

This research shows that elevated CO_2_, climate warming, and management impact shoot and root nitrogen concentrations in different ways in managed pastures. Management (clipping frequency) had the strongest impact on aboveground tissue N concentrations, while the impact of the climate change drivers on shoot N concentration was interactive and varied seasonally. Green leaf N concentrations were decreased by elevated CO_2_ and increased by more frequent clipping. Both warming treatments increased leaf N concentrations under ambient CO_2_ concentrations, but did not significantly alter leaf N concentrations under elevated CO_2_ concentrations. Fine root N concentrations were mostly unaffected by the treatments, although elevated CO2 decreased root N concentration in deeper soil layers. The interactive nature of the climate change drivers through time, as well as the fact that root N concentration response to the treatments was entirely different from aboveground responses, highlights the complexity in predicting plant N nutrition responses to projected climate change.

## Introduction

Long-term responses of plant growth to elevated atmospheric CO_2_ and warming will depend in part on the availability of mineral nutrients and the way in which they are utilized by the plant (Stitt and Krapp 1999; Duval *et al*. 2012). For example, the stimulation of biomass accumulation at elevated CO_2_ is generally less under nitrogen (N) limited than well-fertilized conditions (Norby and Luo 2004; Finzi *et al*. 2006; Gill *et al*. 2006; Reich *et al*. 2006). Leaf and plant N concentrations generally decrease in response to elevated atmospheric CO_2_, and such decreases occur even under conditions of high soil N availability (Cotrufo *et al*. 1998). Thus, decreased tissue N concentration in response to elevated atmospheric CO_2_ is not just due to a N limitation in the growth medium (Poorter *et al*. 1997; Lee *et al*. 2011). Decreased leaf N concentrations have been attributed to either lower N demand as plants utilize rubisco protein more efficiently for photosynthesis under elevated atmospheric CO_2_ concentrations (Drake *et al*. 1997; Stitt and Krapp 1999), or a N-dilution effect as a result of increased carbohydrate production and storage (Coleman *et al*. 1993; Loladze 2002), or diminished ability of plants to assimilate nitrate under high CO_2_ conditions (Rachmilevitch *et al*. 2004; Bloom *et al*. 2010, 2012, 2014). Regarding the negative impact of elevated CO_2_ concentrations on nitrate assimilation, in C_3_ species, suppression of photorespiration by elevated atmospheric CO_2_ concentrations is hypothesized to limit the availability of reductant in the glutamine synthetase and glutamine:2-oxoglutarate amidotransferase cycle. Competition for reductants slows the rate at which nitrate can be converted to glutamate (Bloom 2006), the form of N used in protein synthesis. Since elevated atmospheric CO_2_ and higher air temperatures have opposite effects on photorespiration (Ziska and Bunce 1995), higher temperatures may counteract the suppressing effect of elevated atmospheric CO_2_ on nitrate assimilation in C_3_ plants.

As both warming and elevated atmospheric CO_2_ affect N uptake, translocation and assimilation, they can both be expected to affect tissue N concentrations. Warming applied under ambient atmospheric CO_2_ conditions led to decreased tissue N concentration in both an unfertilized tallgrass prairie (An *et al*. 2005) and a Mediterranean shrubland (Sardans *et al*. 2008). In an interactive CO_2_ × warming experiment, Lilley *et al*. (2001) found no effect of temperature on herbage N concentration in *Phalaris aquatica*, but elevated atmospheric CO_2_ conditions did lower herbage N concentration. Zhou *et al*. (2011) found that elevated atmospheric CO_2_ concentrations led to lower leaf N concentration in *P. arundinacea*, yet higher temperatures led to higher leaf N concentrations overall. These findings on the effect of warming contrast with those of An *et al*. (2005) and Sardans *et al*. (2008) who found lower leaf tissue N concentrations when warming was applied under ambient CO_2_ conditions. Neither Lilley *et al*. (2001) nor Zhou *et al*. (2011) found an interactive effect of warming and atmospheric CO_2_ concentration on leaf N concentration. Anthropogenic global warming is likely greater for night-time minima than for daytime maxima (Karl *et al*. 1991; Vose *et al*. 2005; Gershunov *et al*. 2009), which can also affect plant C balances. For example, Lilley *et al*. (2001) found that *P. aquatica* stores very little carbon (C) as starch, but it maintains high levels of soluble carbohydrates. In this study, soluble carbohydrate concentrations were enhanced by elevated atmospheric CO_2_ but decreased by warming under ambient CO_2_ conditions. Diurnal patterns of warming with higher night-time temperatures, a trend that has been observed in the global temperature records (Vose *et al*. 2005), may have a different effect on plant tissue quality than continuous warming. Temperate steppe grasses responded to nocturnal warming by depleting more stored carbohydrates at night and compensated for this response by enhancing daytime photosynthesis to such a degree (+19.8 %) that the steppe ecosystem switched from being a minor C source to a C sink (Wan *et al*. 2009). Such strong C balance responses to night-time warming will also likely affect tissue N concentrations and C : N ratios.

Changes in tissue N or C concentrations, or both, will affect tissue C : N ratios, with consequences for growth and decomposition, which can then affect litter decomposition rates and C and N cycling if a change in living tissue C : N ratios translates into altered litter C : N ratios. For example, exposure to increasing atmospheric CO_2_ levels along a CO_2_ gradient led to increased plant tissue C : N ratios, greater aboveground N storage in plant parts and increased C : N ratios in soil organic matter in a Texas grassland (Gill *et al*. 2006). In this same experiment, N mineralization rates were decreased, while C mineralization increased under elevated CO_2_ conditions. One possible explanation was that increased litter C : N ratios may have stimulated microbes to start using the more recalcitrant N-rich C fractions in the soil to meet their N demands.

Few studies have reported on the effect of elevated atmospheric CO_2_ on both root C and N concentrations (Nie *et al*. 2013), and data on interactive effects of atmospheric CO_2_ concentration and temperature on root C and N concentration are even rarer (Dieleman *et al*. 2012). The response of tissue nutrient concentrations to CO_2_ and warming is likely to be dependent on the grazing or clipping regime. Ziter and MacDougall (2013) found that grazing enhanced leaf tissue N concentrations while not affecting root N and sugar concentrations. When heavy grazing was compared with moderate grazing, Biondini *et al*. (1998) found that heavy grazing reduced root N concentrations. As grazing removes substantial amounts of biomass from the system and thus interferes with nutrient cycling, pastures are often fertilized.

The objective of this study was to investigate the interactive effects of both atmospheric warming and elevated atmospheric CO_2_ concentration on N concentration and C : N ratio of leaves, roots and litter of *P. aquatica* plantings subjected to two different clipping intensities (infrequent and frequent) to simulate grazing. We reduced the impact of feedbacks due to drought or nutrient limitation by providing ample water and nutrients. Therefore, we report on the direct impacts of both elevated atmospheric CO_2_ and warming on tissue quality in a managed pasture system.

## Methods

### Species description

*Phalaris aquatica* L., previously also known as *P. tuberosa* L., is a highly productive deep-rooted perennial grass originating from the Mediterranean and Middle East and was first introduced as a pasture grass in Australia in 1877 (Oram *et al*. 2009). The common name for *P. aquatica* is ‘phalaris’, although sometimes the name ‘Hardinggras’ is also used (Oram *et al*. 2009). *Phalaris aquatica* is the most widely sown perennial grass in temperate areas of south-eastern Australia. In addition, it is also used in pastures in the USA, South America and New Zealand and to a limited extent in parts of Africa and southern Europe (Oram *et al*. 2009). The cultivar used in this experiment, ‘Holdfast’, is grazing tolerant, winter active and exhibits low summer dormancy. The cultivar ‘Holdfast’ was developed by CSIRO, Canberra, Australia, to have reduced aluminium sensitivity and tolerate acid soils (Oram *et al*. 1993; Oram and Culvenor 1994).

*Phalaris aquatica* is considered an environmental weed in both Australia (Stone 2009) and the Western USA, where it thrives in areas with deep soil and adequate soil moisture (>500 mm rainfall). Due to low seedling vigour, the ability to establish outside cultivation is low, unless bare patches of soil are available (Stone 2009). Once established in natural ecosystems, it can outcompete native species through its ability to form dense clumps with deep root systems that allow it to survive periods of drought (Stone 2009). In general, *P. aquatica* has a high nutrient requirement, especially for N and P, which inhibits its ability to be highly productive outside agricultural situations (Stone 2009). The California Invasive Plant Council rates *P. aquatica* as moderately invasive in California (IPC 2015).

### Temperature gradient tunnels and environmental conditions

The experiment site and tunnel controls are described in Volder *et al*. (2004). Briefly, six transparent ventilated temperature gradient tunnels (TGT, 18 × 1.5 × 1.5 m each) were established on a uniform flat fallow field at Ginninderra Experimental Station (Canberra, Australian Capital Territory, Australia, lat. 35.22°S, long. 149.13°E). During the experiment, hourly averaged ambient temperatures ranged from −5.5 °C (24 August 2002) to 43.6 °C (18 January 2003) with a daily mean of 14.3 °C (Volder *et al*. 2004). Three tunnels were kept at ambient atmospheric CO_2_ concentrations (i.e. no CO_2_ adjustment) and three tunnels were designed to maintain elevated atmospheric CO_2_ concentrations (target: 750 p.p.m.) by injecting CO_2_ into the airstream at two locations. CO_2_ concentrations in the airstream were measured (Model ADC-2000, Analytical Development Co. Ltd, Hoddesdon, UK) at downstream locations every 0.3 s and pulse lengths were adjusted every 1 s accordingly (Volder *et al*. 2004). The daily CO_2_ concentration over the whole experiment in the three elevated atmospheric CO_2_ tunnels was 759 ± 12.6 p.p.m. In the ambient CO_2_ tunnels, the daily average was 403 p.p.m. During the day, the drawdown between the start and the end of the tunnels was ∼7 p.p.m. The tunnels were constructed using a thin aluminium framework with Teflon (Nowoflon ET-film 6235, Nowoflon Kunststoffprodukte GmbH and Co., Siegsdorf, Germany) panels. Radiation energy intercepted by the tunnel structure varied between 20 and 40 %, with the highest proportional interception at midday and in winter (Volder *et al*. 2004).

Within each tunnel, three plant sections (3 × 1.5 × 1.5 m each) were established where the air temperature was either kept at ambient, or a constant warming of +3.0 °C above ambient (constant warming), or +2.2 °C warming above ambient during the day and +4.0 °C warming during the night (high night-time warming). Warming was accomplished mostly by passive solar heating and variable fan speed as air moved through the tunnels during the day and by using a combination of air heaters and drawing in cool air at night (Volder *et al*. 2004). Temperatures were controlled using double-shielded, continuously aspirated thermistors, located 1 m above the surface in each plant section, connected to a Microzone II controller that controlled fan speed and activity of the air heaters. Temperatures were measured every 0.1 s, and fan speed and heater outputs were adjusted every 0.5 s based on the measured temperature differential from the target temperature. Thermistors used for control were independent from the thermistors used to log section air temperatures to ensure data integrity. Daily averaged air temperature warming was +2.9 °C in the high night-time warming treatment and +3.0 °C in the constant warming treatment.

The plots were maintained at non-limiting soil water levels, using overhead irrigation with 16 nozzles per section to ensure even distribution. Soil water content was measured using Theta probes (Delta-T devices Ltd., Cambridge, UK) installed at 5 cm depth in the infrequently clipped plots. In Year 1, soil water content was maintained at 0.32–0.38 m^3^ m^−3^ (77–92 % of field capacity). Starting 25 September 2002, soil water content was reduced to between 0.20 and 0.25 m^3^ m^−3^. Soil water levels were checked daily and when a Theta probe read below 0.20 m^3^ m^−3^, water was applied to all plots until the driest probe read 0.25 m^3^ m^−3^. All sections received the same amount of water. There was always a higher soil moisture content (by 0.02–0.05 m^3^ m^−3^) in the elevated CO_2_ treatment (Volder *et al*. 2004).

Perennial temperate pasture grass (*P. aquatica* cv. Holdfast) was winter-sown at a density of 250 plants m^−2^ using a row spacing of 8 cm on 3 May 2001. Within each temperature treatment, there were two clipping regimes, both at 7 cm above the ground in 1.5 × 1.5 m areas. One regime was clipped two times as often as the other, using visual criteria to decide the time for clipping. The frequently clipped treatment dates were 25 October and 21 November 2001; 8 January, 19 February, 4 April, 1 May, 28 August, 25 September, 6 November, 11 December 2002 and 5 February, 5 March, 8 April and 20–21 May 2003. Plots for the infrequent clipping were clipped every second date. A border zone of 30 cm width was established around each measured plot to avoid edge effects. Both border and measured plot (90 × 90 cm) zones were clipped at each harvest, but border zone material was discarded. Random subsamples (3–6 % of total dry mass) were taken from the bulk sample at each harvest during the harvesting of each plot. The subsamples were divided into standing dead (i.e. litter), green leaf lamina and remainder (sheath, stem and flowers). Sheath, stems and reproductive parts were placed together as most of the stem growth occurs in support of the reproductive parts. All fractions were dried for a week at 80 °C and weighed. Total C and N were determined using a Europa elemental analyser (Sercon, Cheshire, UK). At the final harvest, stubble remaining below the clipping height was harvested from two 20 × 20 cm quadrats placed randomly within each plot. All stubble material including crowns was cleaned free of soil and any roots were discarded. Total C and N were measured as above.

Soil cores to 30 cm depth were collected with 32-mm diameter push tubes from each CO_2_ × temperature × clipping combination on 25 September 2001 (start of treatments), 20 February 2002, 26 September 2002 and 5 March 2003. At the end of the experiment, cores were collected down to 1 m using a hydraulic corer. Cores were cut into 0–10, 10–20 and 20–30 cm segments, with additional segments for the final deeper cores at 50–60 and 80–90 cm. Roots were then washed with distilled water, sieved through a 2-mm sieve, followed by a 0.5-mm sieve (Volder *et al*. 2007) and split into two size classes, fine laterals (first-order roots, less than ∼0.3 mm diameter) and lateral-bearing coarse roots (generally >0.3 mm diameter). Fine laterals were removed from the coarse roots and placed in the fine fraction. All fractions were dried for a week at 80 °C, weighed and total C and N was determined using a Europa elemental analyser (Sercon).

All plots were fertilized three times per year with 100 kg N ha^−1^ per occasion using a slow release fertilizer (Osmocote, Scotts Company, Marysville, OH, USA), which also included 26.7 kg P ha^−1^ and 50.6 kg K ha^−1^. Fertilizer was applied on 28 September 2001; 20 February, 18 June and 29 September 2002 and 6 March 2003.

### Statistical analyses

When necessary to improve homogeneity of variances, data were natural log transformed (Zar 1984). Effects of CO_2_, temperature and clipping frequency were analysed with ANOVA (residual maximum likelihood procedure) using JMP Pro for windows version 10 (SAS Institute, Cary, NC, USA). The design was a split–split plot with CO_2_ level (ambient, 750 p.p.m.) as the main plot factor, warming treatment (+0, +2.2/+4.0 and +3.0 °C) as subplots and clipping frequency (regular, frequent) as randomly assigned sub-subplots within each warming treatment.

## Results

**Figure 1.**
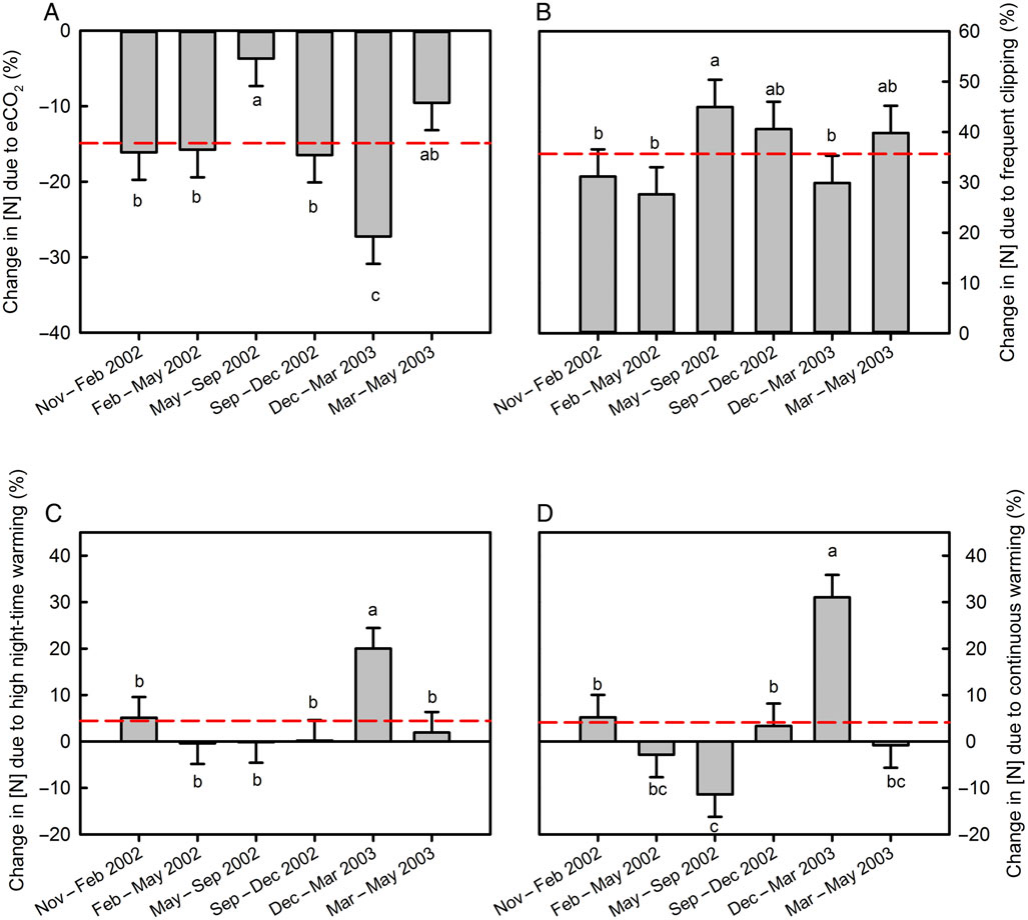
Change in green leaf N concentrations due to (A) increased atmospheric CO_2_ concentration (756 p.p.m.) compared with ambient atmospheric CO_2_ concentration (405 p.p.m.), (B) increased clipping frequency, (C) higher night-time warming compared with ambient air temperatures (+2.0/+4.4 °C above ambient, day/night) and (D) continuous warming above ambient (+3.0 °C), as affected by harvest period. The dashed lines indicate the average response to elevated atmospheric CO_2_ levels (A), increased clipping frequency (B), high night-time warming (C) and continuous warming (D). Data presented are least square means and SEM based on a full model. Different letters indicate statistically significant differences between harvest periods at *P* < 0.05 using Student's *t* LSD test.

Across treatments and harvests, harvested green leaf tissue N concentrations were decreased by 15.6 % under elevated atmospheric CO_2_ concentration (Fig. 1A) and increased by 32.0 % by frequent clipping (Fig. 1B). The influence of elevated CO_2_ and clipping frequency on leaf N concentration varied with harvest period (Fig. 1, Table 1, **see Supporting Information—Tables S1–S3** for tissue C and N concentrations and C : N ratios on each harvest date). The decline in leaf tissue N concentration due to elevated CO_2_ was stronger in spring and summer (November–May and September–March) than during the winter (Fig. 1A, $P_{\mathrm{harvest}\;\mathrm{date}\times CO_{2}}<0.001$), while the effect of clipping frequency was stronger in the winter and fall (May–December, March–May) than in the spring and summer (Fig. 1B, *P*_harvest date × clipping_ < 0.001).

**Table 1.** *P*-values of the effect of atmospheric CO_2_ concentration (CO_2_), warming treatment (*W*), clipping frequency (*C*) and harvest period (*H*) on green leaf N concentration, C concentration and C : N ratio. ^1^Numerator, denominator. ^2^Data were ln transformed prior to analysis. ^3^Missing data prevented analysis of the four-way interaction term. *P* values <0.10 but >0.05 are given in italics, while *P* values <0.05 are given in bold.

	df^1^	Green leaf						Litter					
		N		C		C : N ratio		N^2^		C		C : N ratio	
		*F*	*P*	*F*	*P*	*F*	*P*	*F*	*P*	*F*	*P*	*F*	*P*
CO_2_	1,4	130	**<0.001**	0.99	0.375	98.1	**<0.001**	0.90	0.385	6.39	*0.064*	0.22	0.651
Warming (*W*)	2,8	0.83	0.470	5.88	**0.027**	0.38	0.694	4.25	**0.047**	8.61	**0.010**	0.23	0.798
CO_2_ × *W*	2,8	5.17	**0.036**	0.48	0.633	3.48	*0.082*	2.44	0.140	3.37	*0.087*	1.80	0.219
Clipping frequency (*C*)	1,12	196	**<0.001**	0.07	0.799	160	**<0.001**	55.0	**<0.001**	2.10	0.171	29.6	**<0.001**
*C* × CO_2_	1,12	0.16	0.691	0.07	0.797	2.32	0.153	0.00	0.947	0.21	0.652	0.48	0.498
*C* × *W*	2,12	0.49	0.615	2.45	0.128	1.24	0.325	0.24	0.788	1.72	0.217	0.18	0.838
*C* × *W* × CO_2_	2,12	0.55	0.584	0.41	0.674	0.70	0.518	0.42	0.668	0.68	0.523	0.52	0.607
Harvest (*H*)	5,120	79.5	**<0.001**	61.1	**<0.001**	39.8	**<0.001**	6.25	**<0.001**	15.3	**<0.001**	0.98	0.435
*H* × CO_2_	5,120	6.39	**<0.001**	1.35	0.249	5.00	**<0.001**	1.05	0.392	1.38	0.236	0.33	0.893
*H* × *W*	10,120	4.43	**<0.001**	2.89	**0.003**	4.05	**<0.001**	1.15	0.334	1.02	0.434	0.66	0.761
*H* × CO_2_ × *W*	10,120	0.75	0.662	1.22	0.286	0.91	0.528	1.17	0.317	1.20	0.296	0.67	0.752
*H* × Clip	5,120	5.17	**<0.001**	14.2	**<0.001**	2.02	*0.080*	16.7	**<0.001**	11.9	**<0.001**	13.4	**<0.001**
*H* × Clip × CO_2_	5,120	*2.05*	*0.075*	0.19	0.964	0.85	0.515	0.47	0.796	1.46	0.207	0.41	0.839
*H* × Clip × *W*	10,120	0.78	0.652	1.48	0.156	0.61	0.806	1.51	0.143	1.47	0.157	1.07	0.392
*H* × Clip × CO_2_ × *W*	10,120	0.42	0.936	1.07	0.393	0.408	0.941		NA^3^		NA^3^		NA^3^
Model *r*^2^			0.898		0.779		0.874		0.649		0.620		0.621
Model *P*			<0.001		<0.001		<0.001		<0.001		<0.001		<0.001

**Figure 2.**
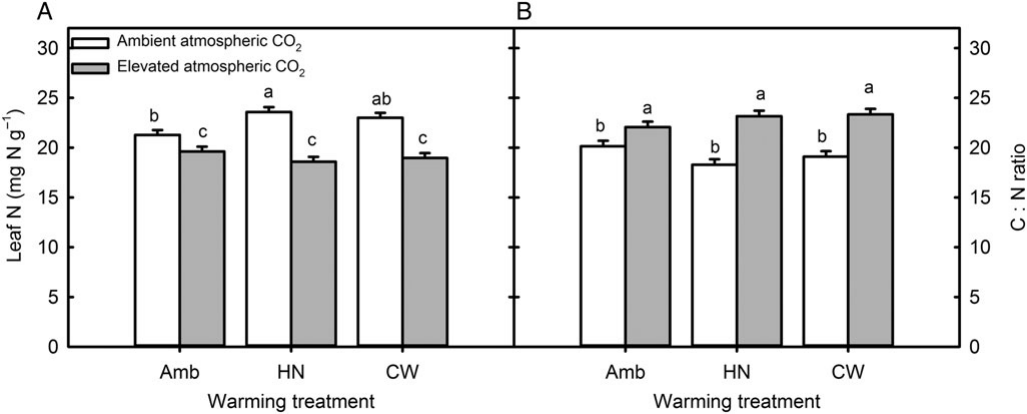
Effect of warming treatment, Amb = ambient temperature, HN = +2.2/+4.0 °C (day/night warming), CW = +3.0 °C continuous warming and atmospheric CO_2_ concentration (ambient, 405 p.p.m., and elevated, 756 p.p.m.) on (A) green leaf N concentration and (B) green leaf C : N ratio. Data presented are least square means and SEM averaged across harvests and clipping frequencies. Different letters indicate statistically significant differences at *P* <0.05 using Student's *t* LSD test.

Warming was without any overall effect on green leaf N concentration when averaged across all the other treatments and harvest times (Table 1). However, there were strong interactive effects of warming with CO_2_ and harvest period. Averaged across CO_2_ treatments, both warming treatments greatly increased leaf N concentration in the second summer (*P*_harvest × warm_ < 0.001, December–March 2003, Fig. 1C and D). In contrast, continuous warming decreased leaf N concentration in comparison with ambient temperatures in the winter (May–September 2002, Fig. 1C). When averaged across all dates and clipping frequencies, warming significantly increased leaf N concentrations under ambient CO_2_, but under elevated CO_2_, leaf N concentration was not affected by warming (Fig. 2A). The negative effect of increased atmospheric CO_2_ concentration on leaf N concentration was substantially enhanced by both warming treatments, from 7.6 % decrease under ambient temperature to 17.5 and 21.4 % decrease under continuous and high night-time warming $\left(P_{CO_{2}\times \mathrm{warm}}=0.036\right)$.

**Figure 3.**
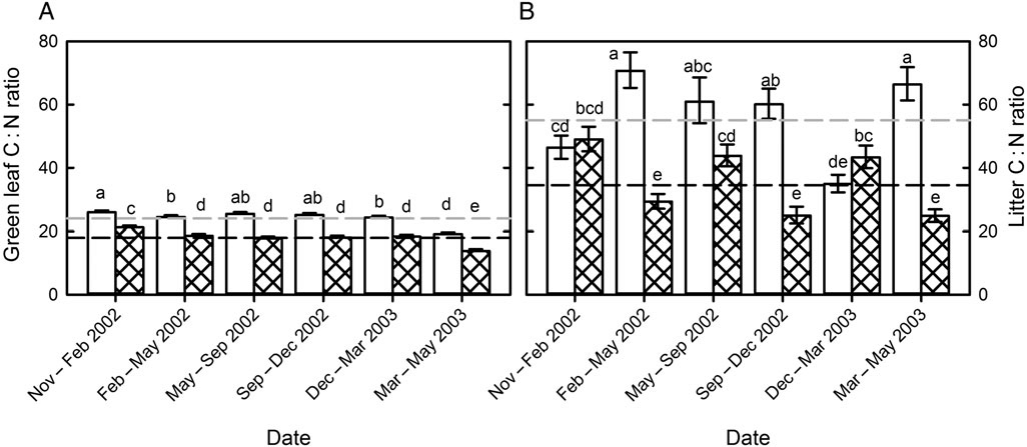
Effect of clipping frequency (open bar = infrequent, hashed bar = frequent) on C : N ratios of (A) green leaf material and (B) litter, through time averaged across CO_2_ and warming treatments. Dashed line indicates overall mean across dates, CO_2_ and warming treatment for each clipping frequency, infrequent (grey) and frequent (black). Different letters indicate statistically significant differences between C : N ratios at *P* < 0.05 using Student's *t* LSD test.

Elevated CO_2_ increased leaf C : N ratios, particularly under the high night-time warming scenario (‘HN’, $P_{CO_{2}\times \mathrm{warming}}=0.053$, from 18.0 to 22.9, Fig. 2B). Changes in leaf C : N ratio in response to elevated CO_2_ reflect changes in leaf N concentration. There was no relationship between changes in C : N ratio and changes in C concentration in response to elevated CO_2_. Increased clipping frequency decreased green leaf C : N ratios by an average of 24 %, from 23.6 to 17.9 (Fig. 3A, *P* < 0.001).

**Figure 4.**
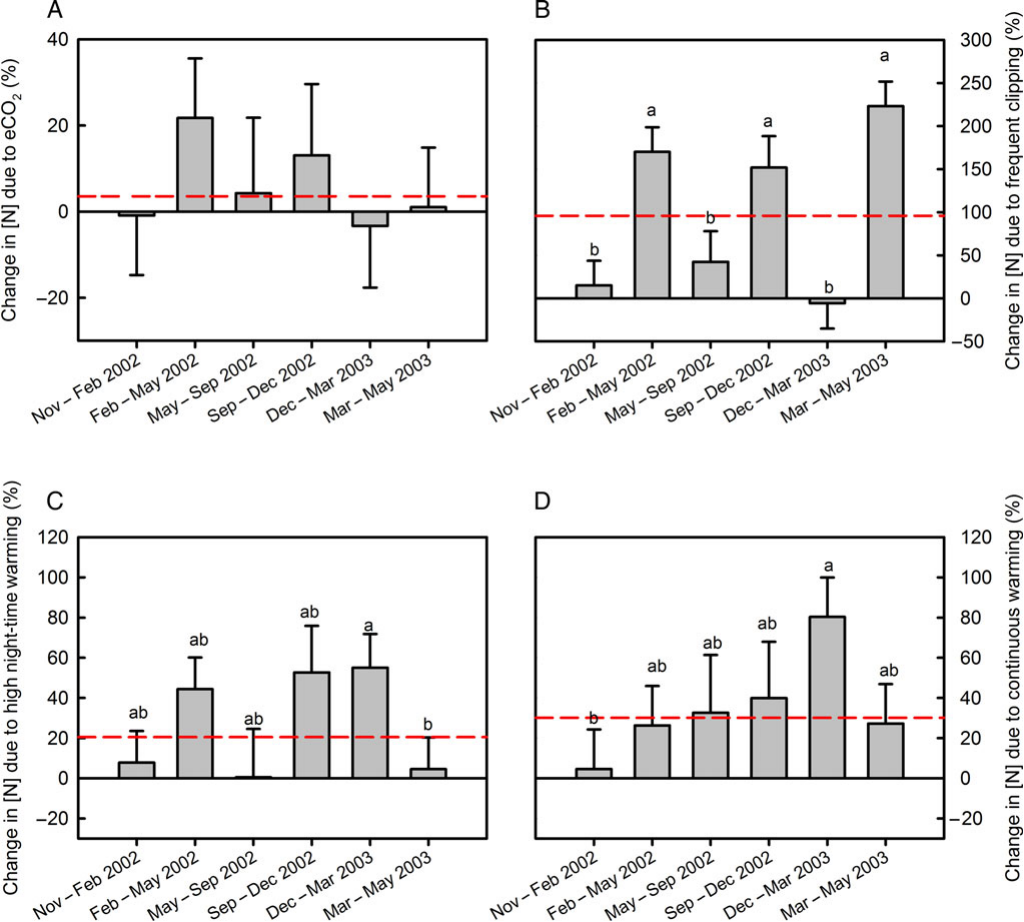
Percent change in litter N concentrations due to (A) increased atmospheric CO_2_ concentration (756 p.p.m.) compared with ambient atmospheric CO_2_ concentration (405 p.p.m.), (B) increased clipping frequency, (C) higher night-time warming compared with ambient air temperatures (+2.0/+4.4 °C above ambient, day/night) and (D) continuous warming above ambient (+3.0 °C), as affected by harvest period. The dashed lines indicate the average response to elevated atmospheric CO_2_ levels (A), increased clipping frequency (B), high night-time warming (C) and continuous warming (D). Data presented are least square means and SEM based on a full model. Different letters indicate statistically significant differences between harvest periods at *P* < 0.05 using Student's *t* LSD test.

**Figure 5.**
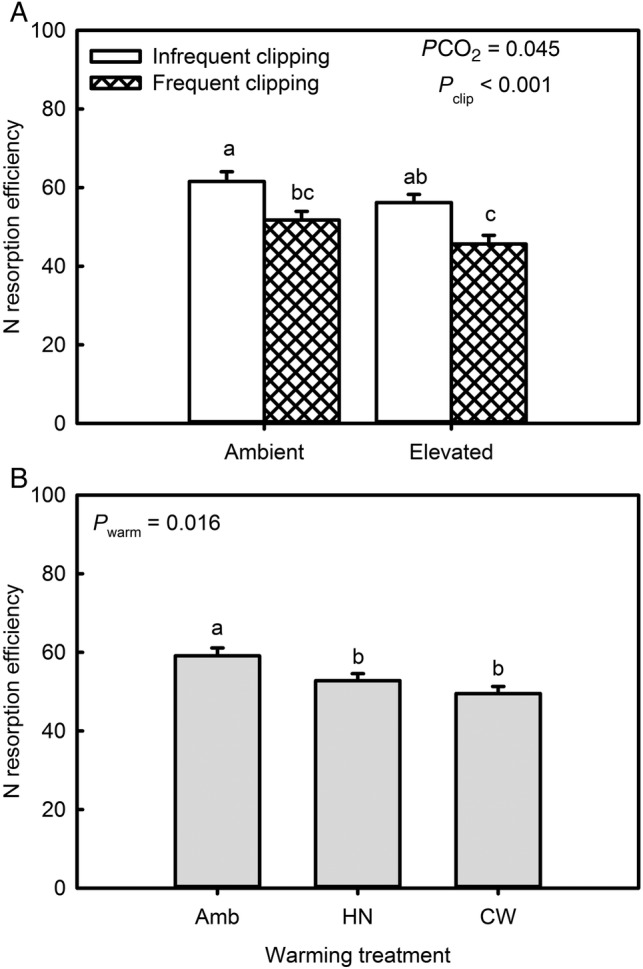
(A) Effect of clipping frequency and atmospheric CO_2_ concentration on N resorption efficiency. (B) Effect of warming treatment on N resorption efficiency, Amb = ambient, HN = +2.2 °C/+4.0 °C day/night warming, CW = +3.0 °C continuous warming. Different letters indicate statistically significant differences at *P* < 0.05 using Student's *t* LSD test.

Litter (i.e. standing dead leaf) N concentrations were unaffected by atmospheric CO_2_ concentration (Table 1 and **see Supporting Information—Table S1**). There was a marginally significant warming effect (Table 1, *P*_warming_ = 0.047) where litter N concentration averaged across harvests was higher under a continuous warming scenario (9.1 mg g^−1^) than under ambient warming (7.6 mg g^−1^). The average of the two warming treatments increased litter N concentration by 25.3 % (Fig. 4C and D). Litter N concentration was most affected by clipping frequency; increased clipping frequency increased litter N concentrations by an average of 95.9 % (Fig. 4B). Consequently, frequent clipping reduced litter C : N ratio from 55.0 to 34.5 averaged across harvests and CO_2_ and warming treatments (Fig. 3B and **see Supporting Information—Table S3**). Nitrogen resorption efficiency, calculated as [100 × (green leaf N concentration − litter N concentration)/green leaf N concentration], was decreased from 56.7 % at ambient atmospheric CO_2_ concentration to 50.9 % under elevated CO_2_ when averaged across warming and clipping treatments (Fig. 5A). Increased clipping frequency decreased N resorption efficiency from 58.9 % in infrequently clipped plots to 48.7 % in frequently clipped plots when averaged across warming and CO_2_ treatments (Fig. 5A). Averaged across clipping and CO_2_ treatment, both warming treatments reduced N resorption efficiency from 59.1 % in the unwarmed treatment to 52.8 and 49.5 % in the high night-time and continuously warmed treatments, respectively.

**Figure 6.**
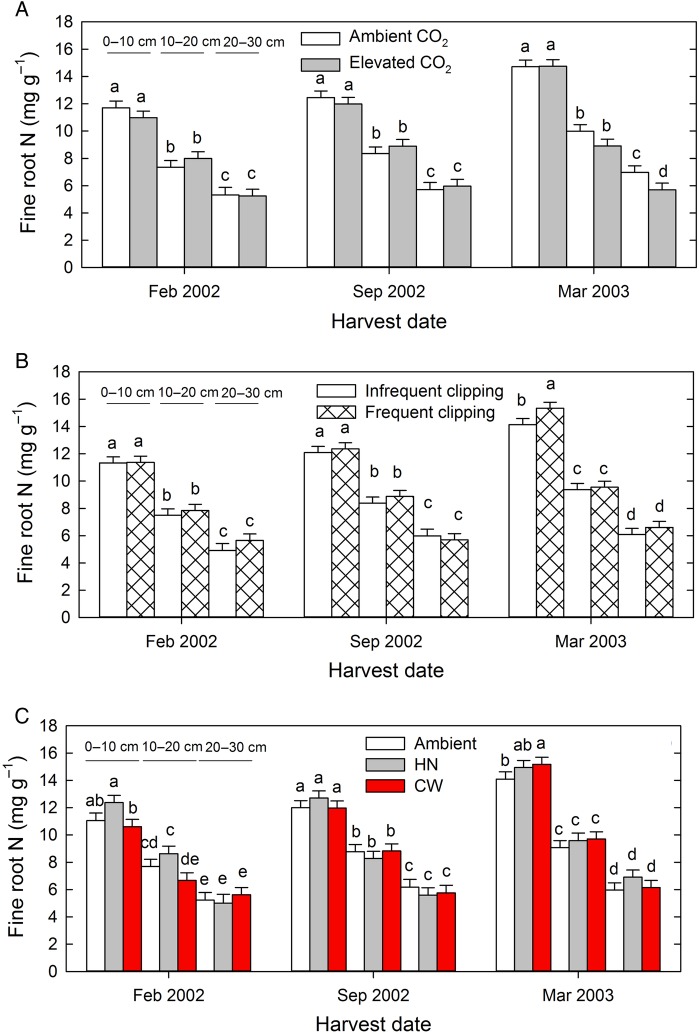
Effect of atmospheric CO_2_ concentration (A), clipping frequency (B) and warming treatment (C) on fine root N concentrations at three harvest dates and three depths. HN = +2.2/+4.0 °C day/night, CW = +3.0 °C continuous warming. Different letters indicate statistically significant differences within a harvest date at *P* < 0.05 using Student's *t* LSD test.

**Figure 7.**
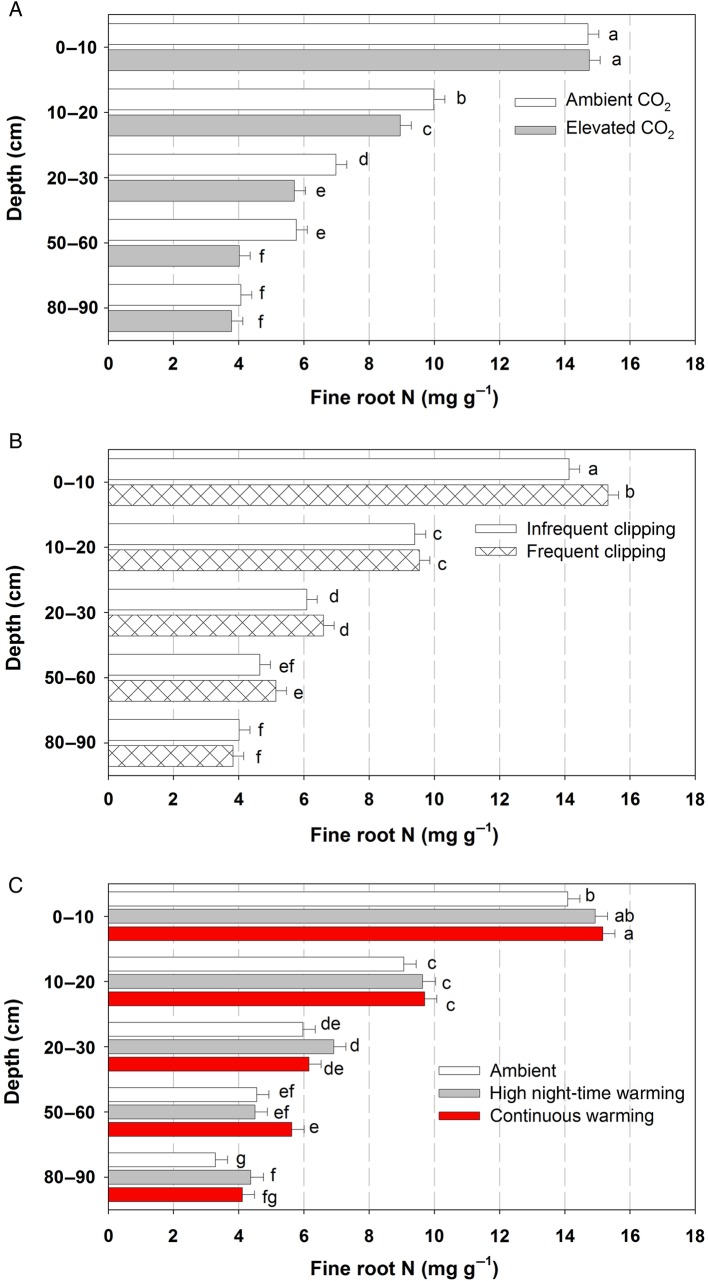
Effect of (A) atmospheric CO_2_ concentration, (B) clipping frequency and (C) warming treatment on fine root N concentration at five depths at the end of the experiment (March 2003). Treatments started in September 2001. Data are the least square means + SEM across treatments. Different letters indicate statistically significant differences at *P* < 0.05 using Student's *t* LSD test.

Fine root N concentrations decreased strongly with depth in the soil on all three harvest dates (Fig. 6, Table 2). Elevated atmospheric CO_2_ had no systematic effect on fine root N concentration, although there was a small significant decline under high CO_2_ in March 2003, in the 20–30 cm soil depth (Fig. 6A). Increased clipping frequency had no overall effect on fine root N concentration but did increase fine root N concentration in just the 0–10 cm soil layer in March 2003 (Fig. 6B). Overall, averaged across CO_2_, clipping frequency and harvests, warming was without effect on root N concentration. However, small effects of warming varied with depth and date and were limited to the shallower (0–10 and 10–20 cm) soil depths (Fig. 6C). In February 2002, high night-time warming led to slightly increased fine root N concentration compared with continuous warming in the 0–10 and 10–20 cm soil depths, while in March 2003, continuous warming led to higher fine root N concentrations in the continuous warming treatment compared with ambient warming in the 0–10 cm soil depth (Fig. 6C). At the final harvest (March 2003), where we also collected deeper soil cores, fine root N concentration decreased further with soil depth below 50 cm. There was a strong atmospheric CO_2_ concentration × depth interaction effect $(P_{CO_{2}\times \mathrm{depth}}=0.009$, Table 2) where elevated atmospheric CO_2_ levels reduced fine root N concentrations at the 10–20, 20–30 and 50–60 cm core depths, but not in the 0–10 and 80–90 cm core depths (Fig. 7). There were no additional effects of clipping frequency or air warming on fine root N of roots growing below 50 cm soil depth (Fig. 7B and C).

**Table 2.** *P*-values of the effect of atmospheric CO_2_ concentration (CO_2_), warming treatment (*W*), clipping frequency (*C*) and soil depth (*D*) on fine root N and C concentrations, and C : N ratio through time. ^1^Depth classes also included 50–60 and 80–90 cm. ^2^Numerator, denominator. ^3^Data were ln transformed prior to analysis.

Treatment	February 2002 (summer)							September 2002 (early spring)							March 2003 (early fall)^1^						
	df^2^	N		C		C : N^3^		df	N		C		C : N^3^		df	N		C		C : N^3^	
		*F* ratio	*P*	*F* ratio	*P*	*F* ratio	*P*		*F* ratio	*P*	*F* ratio	df	*F* ratio	*P*		*F* ratio	*P*	*F* ratio	*P*	*F* ratio	*P*
CO_2_	1,4	0.01	0.942	1.01	0.370	0.64	0.466	1,4	0.03	0.883	3.63	0.127	0.69	0.453	1,4	6.93	*0.059*	0.62	0.475	7.42	*0.054*
Warming (*W*)	2,8	*3.22*	*0.096*	0.76	0.500	2.45	0.166	2,8	0.10	0.906	0.02	0.976	0.96	0.959	2,8	4.46	**0.049**	1.19	0.352	3.20	*0.095*
CO_2_ × *W*	2,8	1.08	0.386	0.01	0.987	0.58	0.589	2,8	0.70	0.527	0.47	0.640	0.60	0.602	2,8	0.30	0.746	0.32	0.737	0.12	0.886
Clipping (*C*)	1,12	1.31	0.275	*3.84*	*0.076*	0.03	0.866	1,12	0.11	0.742	0.45	0.515	0.91	0.915	1,12	2.37	0.150	8.54	**0.013**	22.5	**<0.001**
CO_2_ × Clip	1,12	2.39	0.148	0.05	0.825	1.89	0.197	1,12	0.04	0.852	0.21	0.656	0.73	0.734	1,12	1.63	0.226	0.13	0.730	4.50	*0.055*
*W* × Clip	2,12	0.53	0.602	0.40	0.681	0.10	0.910	2,12	0.41	0.670	0.23	0.799	0.45	0.448	2,12	0.41	0.673	1.96	0.182	2.73	0.105
CO_2_ × *W* × Clip	2,12	1.24	0.324	2.08	0.171	0.10	0.910	2,12	0.15	0.859	0.28	0.762	0.48	0.477	2,12	2.27	0.146	0.06	0.940	3.01	*0.087*
Depth (*D*)	2,44	78.1	**<0.001**	3.82	**0.029**	69.5	**<0.001**	2,46	135	**<0.001**	28.9	**<0.001**	47.8	**<0.001**	2,95	514	**<0.001**	16.5	**<0.001**	427	**<0.001**
*D* × CO_2_	2,44	1.07	0.351	0.28	0.758	1.00	0.377	2,46	1.00	0.375	0.37	0.695	1.12	0.335	2,95	3.59	**0.009**	3.62	**0.009**	3.52	**0.010**
*D* × *W*	4,44	1.40	0.251	1.40	0.249	0.33	0.858	4,46	0.84	0.507	1.64	0.179	0.660	0.665	4,95	1.23	0.291	1.95	*0.062*	1.03	0.420
*D* × CO_2_ × *W*	4,44	0.75	0.566	2.04	0.105	0.66	0.627	4,46	0.79	0.537	1.07	0.381	0.48	0.752	4,95	0.75	0.645	1.41	0.202	1.35	0.228
*D* × *C*	2,44	0.39	0.676	0.70	0.504	0.38	0.683	2,46	0.56	0.576	0.86	0.432	1.38	0.262	2,95	1.74	0.147	2.36	*0.059*	1.31	0.273
*D* × CO_2_ × *C*	2,44	0.39	0.681	3.53	**0.038**	1.83	0.175	2,46	0.79	0.459	2.74	*0.075*	1.94	0.156	2,95	0.98	0.422	0.73	0.574	0.71	0.584
*D* × *W* × *C*	4,44	0.95	0.445	0.70	0.599	0.61	0.656	4,46	0.40	0.807	4.84	**0.002**	2.32	*0.071*	4,95	0.54	0.822	0.60	0.774	1.00	0.442
*D* × CO_2_ × *W* × *C*	4,44	0.21	0.932	0.56	0.691	0.84	0.506	4,46	0.64	0.640	1.32	0.277	0.06	0.994	4,95	1.55	0.152	0.63	0.754	1.02	0.425
Model *r*^2^			0.721		0.448		0.741			0.854		0.681		0.716			0.955		0.566		0.948
Model *P*			<0.001		<0.001		<0.001			<0.001		<0.001		<0.001			<0.001		<0.001		<0.001

## Discussion

Across the three tissues examined, green leaf, litter and fine root, green leaves had the highest tissue N concentration and were the most responsive to elevated atmospheric CO_2_ concentration and warming. Elevated atmospheric CO_2_ concentration generally reduced tissue N concentrations while the effect of warming depended on the CO_2_ concentration. Of the three treatments, frequent clipping had the greatest overall impact on leaf and litter N concentrations, with increased clipping frequency strongly increasing tissue N concentrations. Because treatment impacts varied by tissue type, we will discuss treatment effects by tissue type.

### Green leaf

More frequent clipping strongly increased green leaf N concentration by an average of 33 % across the other treatments and harvest dates (Fig. 1B). This was expected because the frequently clipped plants have shoot tissue that is about half the age of shoot tissue of infrequently clipped plants. Younger tissue generally has higher N concentrations (Field 1983). Just why the magnitude of this clipping-frequency effect was greater in the slower growing winter months (May–September) is not clear.

Despite being well fertilized, elevated CO_2_ concentration decreased green leaf N concentrations in these field grown C_3_ grasses. That result is consistent with the literature (Cotrufo *et al*. 1998). The mean decrease in leaf N concentration in response to elevated CO_2_ was 15 % (Fig. 1A), which is similar to the 17 % decrease in N concentration for non-woody C_3_ species reported by Cotrufo *et al*. (1998). The magnitude of lower leaf N concentration in response to elevated atmospheric CO_2_ varied seasonally with a much smaller response to elevated CO_2_ in the slower growing winter season. The biomass production response to elevated atmospheric CO_2_ in this same experiment was also smaller during the cold months (Volder *et al*. 2004), consistent with the notion (Coleman *et al*. 1993; Loladze 2002) that dilution of leaf N concentration by enhanced growth in response to elevated CO_2_ plays a role at least in part. However, the strong relationship between changes in leaf N concentration and changes in leaf C : N ratio, as well as the lack of a relationship between changes in leaf C concentration and changes in leaf C : N ratio, supports the idea that effects of elevated CO_2_ on green leaf tissue C : N ratio are mostly driven by the effects of CO_2_ on leaf N concentrations rather than C accumulation in response to elevated CO_2_ (Gifford *et al*. 2000).

Atmospheric warming treatments, when averaged across all harvests, increased green leaf N concentrations of plants grown under ambient atmospheric CO_2_ concentrations by 11.4 and 8.6 % for the higher night and continuous warming treatments, respectively (Fig. 2A, open bars). However, warming had no significant effect on leaf N concentrations of the swards grown under elevated CO_2_ (Fig. 2A, closed bars). The effects of CO_2_ concentration and warming on leaf N concentrations were not simply additive; warming of ambient CO_2_ plants enhanced green leaf N concentrations by ∼10 %, elevated CO_2_ decreased leaf N concentrations by 7.6 %, while combined warming and elevated CO_2_ decreased green leaf N concentrations by ∼11 % (Fig. 2A). Non-additivity of warming and CO_2_ responses, with the response to CO_2_ dominating, is also consistent with a meta-analysis of leaf tissue N in six temperature × CO_2_ manipulation studies by Dieleman *et al*. (2012). The increase in tissue N in response to warming at ambient atmospheric CO_2_ levels is consistent with the idea that shoot nitrate assimilation depends on photorespiration (Rachmilevitch *et al*. 2004; Bloom *et al*. 2010, 2012). Under conditions where photorespiration is enhanced (i.e. ambient CO_2_ and warming), nitrate assimilation is stimulated, while under conditions where photorespiration is repressed (i.e. high atmospheric CO_2_ conditions), increasing temperatures will not affect tissue N concentrations. This suggests that the impact of future climate warming on tissue quality and N cycling cannot be predicted based on warming experiments alone.

Our finding that green leaf tissue N concentration increased in response to warming under ambient atmospheric CO_2_ concentrations differs from An *et al*. (2005), who found that warming decreased tissue N concentrations in five grassland species. An *et al*. (2005) observed the effects of warming after 1 year of treatment, whereas in our study, warming did not have a statistically significant effect on green leaf N concentrations until well into the second season of treatments. Thus, the effect of warming may not be as immediate and is more subtle than the effects of elevated atmospheric CO_2_ and clipping frequency.

In contrast to the effects of combined warming and atmospheric CO_2_ on leaf N concentration, there was no interaction between clipping frequency and CO_2_ concentration or between clipping frequency and warming treatment. This suggests that the effects of clipping management are additive to the effects of the climate change drivers. Our earlier findings on aboveground biomass production (Volder *et al*. 2004) and new root production (Volder *et al*. 2007) showed non-additivity, where the effect of elevated CO_2_ was dependent on clipping frequency. Thus, as expected, whether effects of climate change drivers are additive or not depends on the response variable measured and the management regimen of the ecosystem (Dieleman *et al*. 2012; Xu *et al*. 2013).

### Standing dead and leaf litter

The decreases in leaf N concentration due to elevated atmospheric CO_2_ concentrations (Figs 1A and 2A) did not translate into reduced standing dead leaf litter N concentration or increased litter C : N ratios (Fig. 4A). Neither litter N concentration nor litter C : N ratio were significantly affected by elevated CO_2_ (Table 1). Increased clipping frequency strongly decreased C : N ratios of both leaf (from 24.0 to 18.0) and litter (from 55.0 to 34.5, Fig. 3). Frequent clipping increased green leaf N concentration and also reduced N resorption efficiency from 59.2 to 53.6 %, thus leaving a greater amount of N per gram litter biomass. As more N is left in senescing tissues of frequently clipped vegetation and litter C : N ratios are reduced, it is possible that frequent clipping would speed up the rate of mineralization of litter N from a given amount of litter added to the soil (Booth *et al*. 2005), if soil moisture and soil temperature remain similar. However, whether that would lead to actual increased N availability in soils of frequently cut swards also depends on the total amount of litter returned to the soil. Previous published results (Volder *et al*. 2004) showed that frequent clipping strongly decreased biomass production, and thus the total amount of N returned to the soil from litter may be reduced, even if mineralization rates are increased because of decreased litter C : N ratios.

### Roots

Root N concentrations were very strongly affected by soil depth; the concentrations in fine roots at 80–90 cm depth were less than a third of those in the top 10 cm (Fig. 7). Effects of warming, CO_2_ and clipping frequency were minor in comparison with the depth effect. These minor effects of elevated CO_2_ concentration and warming at depth were most evident at the final harvest. Although most CO_2_ responses occurred in roots below 10 cm soil depth in our experiment, others have found negative impacts of elevated CO_2_ concentrations on root N concentration in shallow (<10 cm) soil layers in grassland systems (Kitchen *et al*. 2009). The lack of a clipping effect on root N concentration was surprising given that increased clipping frequency increased root turnover rate (Volder *et al*. 2007), which would lead to a younger root system on average. Younger roots have been shown to have higher rates of nitrate uptake and higher tissue N concentrations (Volder *et al*. 2005); however, the proportional change in average root age may not have been large enough to affect the average tissue N concentration in our bulk samples.

Often root research is limited to the top soil layer because that is the zone where generally >50 % of root length occurs (Schenk and Jackson 2002). However, our data suggest that when evaluating the impact of climate on root parameters, some major changes in tissue N concentrations may be occurring deeper in the soil profile. It is important to note that our experiment involved a managed pasture grass system where water and nutrients were supplied at high levels—soil water content was kept at 20 % or higher (Volder *et al*. 2004), and the plots were fertilized three times per year at a rate of 100 kg N ha^−1^ per occasion. Thus, plant responses in our system were mostly decoupled from soil system feedbacks (Type I system, Körner 2006) when compared with other climate change experiments in grasslands, which generally take place in systems where water and/or nutrients are limited (Morgan *et al*. 2004).

## Conclusions

While increasing atmospheric CO_2_ concentration decreased green leaf N concentrations considerably, this was not propagated to leaf litter, as leaf litter N concentration was unaffected by elevated CO_2_. Atmospheric warming increased green leaf N under ambient CO_2_ but did not significantly affect leaf N concentration at elevated CO_2_ concentration. The increase in leaf N concentration under warming at ambient CO_2_ was reflected in increased litter N concentration. For fine roots, elevated CO_2_ tended to decrease N concentration (*P* = 0.059) and increase C : N ratio by the end of the experiment with the magnitude of the effect increasing deeper in the soil. The effects of continuous uniform warming were similar to differential day/night warming.

In general, the non-additivity of CO_2_, warming and management treatment effects with unexplained time variability of specific interactive effects that are exhibited in this data set presents considerable problems for predicting long-term climate change impacts on pasture ecophysiology by either rules of thumb or simulation modelling.

## Sources of Funding

This work was funded by the Cooperative Research Centre for Greenhouse Accounting and internal funding from CSIRO Division of Plant Industry, Australia.

## Contributions by the Authors

A.V. collected and processed samples, performed data analysis and wrote the manuscript. R.M.G. and J.R.E. collected samples and co-wrote the manuscript.

## Conflict of Interest Statement

None declared.

## Supporting Information

The following additional information is available in the online version of this article –

**Table S1.** Tissue N concentrations (mg N g^−1^) through time as affected by atmospheric CO_2_ concentration, warming treatment, ambient, higher night-time warming and continuous warming, and clipping frequency.

**Table S2.** Tissue C concentrations (g C g^−1^) through time as affected by atmospheric CO_2_ concentration, warming treatment, ambient, higher night-time warming and continuous warming, and clipping frequency.

**Table S3.** Tissue C : N ratios through time as affected by atmospheric CO_2_ concentration, warming treatment, ambient, higher night-time warming and continuous warming, and clipping frequency.

Additional Information

## References

[PLV094C1] An Y, Wan S, Zhou X, Subedar AA, Wallace LL, Luo Y 2005 Plant nitrogen concentration, use efficiency, and contents in a tallgrass prairie ecosystem under experimental warming. Global Change Biology 11:1733–1744. 10.1111/j.1365-2486.2005.01030.x

[PLV094C2] Biondini ME, Patton BD, Nyren PE 1998 Grazing intensity and ecosystem processes in a northern mixed-grass prairie, USA. Ecological Applications 8:469–479. 10.1890/1051-0761(1998)008[0469:GIAEPI[2.0.CO;2

[PLV094C3] Bloom AJ 2006 Rising carbon dioxide concentrations and the future of crop production. Journal of the Science of Food and Agriculture 86:1289–1291. 10.1002/jsfa.2502

[PLV094C4] Bloom AJ, Burger M, Asensio JSR, Cousins AB 2010 Carbon dioxide enrichment inhibits nitrate assimilation in wheat and *Arabidopsis*. Science 328:899–903. 10.1126/science.1186440 20466933

[PLV094C5] Bloom AJ, Asensio JSR, Randall L, Rachmilevitch S, Cousins AB, Carlisle EA 2012 CO_2_ enrichment inhibits shoot nitrate assimilation in C_3_ but not C_4_ plants and slows growth under nitrate in C_3_ plants. Ecology 93:355–367. 10.1890/11-0485.1 22624317

[PLV094C6] Bloom AJ, Burger M, Kimball BA, Pinter PJ Jr 2014 Nitrate assimilation is inhibited by elevated CO_2_ in field-grown wheat. Nature Climate Change 4:477–480.

[PLV094C7] Booth MS, Stark JM, Rastetter E 2005 Controls on nitrogen cycling in terrestrial ecosystems: a synthetic analysis of literature data. Ecological Monographs 75:139–157. 10.1890/04-0988

[PLV094C8] Coleman JS, McConnaughay KDM, Bazzaz FA 1993 Elevated CO_2_ and plant nitrogen-use: is reduced tissue nitrogen concentration size-dependent? Oecologia 93:195–200. 10.1007/BF00317671 28313607

[PLV094C9] Cotrufo MF, Ineson P, Scott A 1998 Elevated CO_2_ reduces the nitrogen concentration of plant tissues. Global Change Biology 4:43–54. 10.1046/j.1365-2486.1998.00101.x

[PLV094C10] Dieleman WIJ, Vicca S, Dijkstra FA, Hagedorn F, Hovenden MJ, Larsen KS, Morgan JA, Volder A, Beier C, Dukes JS, King J, Leuzinger S, Linder S, Luo Y, Oren R, De Angelis P, Tingey D, Hoosbeek MR, Janssens IA 2012 Simple additive effects are rare: a quantitative review of plant biomass and soil process responses to combined manipulations of CO_2_ and temperature. Global Change Biology 18:2681–2693. 10.1111/j.1365-2486.2012.02745.x 24501048

[PLV094C11] Drake BG, Gonzàlez-Meler MA, Long SP 1997 More efficient plants: a consequence of rising atmospheric CO_2_? Annual Review of Plant Physiology and Plant Molecular Biology 48:609–639. 10.1146/annurev.arplant.48.1.609 15012276

[PLV094C12] Duval BD, Blankinship JC, Dijkstra P, Hungate BA 2012 CO_2_ effects on plant nutrient concentration depend on plant functional group and available nitrogen: a meta-analysis. Plant Ecology 213:505–521. 10.1007/s11258-011-9998-8

[PLV094C13] Field C 1983 Allocating leaf nitrogen for the maximization of carbon gain: leaf age as a control on the allocation program. Oecologia 56:341–347. 10.1007/BF00379710 28310214

[PLV094C14] Finzi AC, Moore DJP, DeLucia EH, Lichter J, Hofmockel KS, Jackson RB, Kim HS, Matamala R, McCarthy HR, Oren R, Pippen JS, Schlesinger WH 2006 Progressive nitrogen limitation of ecosystem processes under elevated CO_2_ in a warm-temperate forest. Ecology 87:15–25. 10.1890/04-1748 16634293

[PLV094C15] Gershunov A, Cayan DR, Iacobellis SF 2009 The great 2006 heat wave over California and Nevada: signal of an increasing trend. Journal of Climate 22:6181–6203. 10.1175/2009JCLI2465.1

[PLV094C16] Gifford RM, Barrett DJ, Lutze JL 2000 The effects of elevated [CO_2_] on the C : N and C : P mass ratios of plant tissues. Plant and Soil 224:1–14. 10.1023/A:1004790612630

[PLV094C17] Gill RA, Anderson LJ, Polley HW, Johnson HB, Jackson RB 2006 Potential nitrogen constraints on soil carbon sequestration under low and elevated atmospheric CO_2_. Ecology 87:41–52. 10.1890/04-1696 16634295

[PLV094C18] IPC California. 2015 http://www.cal-ipc.org/ip/management/plant_profiles/Phalaris_aquatica.php (23 June 2015).

[PLV094C19] Karl TR, Kukla G, Razuvayev VN, Changery MJ, Quayle RG, Heim RR, Easterling DR, Fu CB 1991 Global warming: evidence for asymmetric diurnal temperature change. Geophysical Research Letters 18:2253–2256. 10.1029/91GL02900

[PLV094C20] Kitchen DJ, Blair JM, Callaham MA Jr 2009 Annual fire and mowing alter biomass, depth distribution, and C and N content of roots and soil in tallgrass prairie. Plant and Soil 323:235–247. 10.1007/s11104-009-9931-2

[PLV094C21] Körner C 2006 Plant CO_2_ responses: an issue of definition, time and resource supply. New Phytologist 172:393–411. 10.1111/j.1469-8137.2006.01886.x 17083672

[PLV094C22] Lee TD, Barrott SH, Reich PB 2011 Photosynthetic responses of 13 grassland species across 11 years of free-air CO_2_ enrichment is modest, consistent and independent of N supply. Global Change Biology 17:2893–2904. 10.1111/j.1365-2486.2011.02435.x

[PLV094C23] Lilley JM, Bolger TP, Peoples MB, Gifford RM 2001 Nutritive value and the nitrogen dynamics of *Trifolium subterraneum* and *Phalaris aquatica* under warmer, high CO_2_ conditions. New Phytologist 150:385–395. 10.1046/j.1469-8137.2001.00101.x

[PLV094C24] Loladze I 2002 Rising atmospheric CO_2_ and human nutrition: toward globally imbalanced plant stoichiometry? Trends in Ecology and Evolution 17:457–461. 10.1016/S0169-5347(02)02587-9

[PLV094C25] Morgan JA, Pataki DE, Körner C, Clark H, Del Grosso SJ, Grünzweig JM, Knapp AK, Mosier AR, Newton PCD, Niklaus PA, Nippert JB, Nowak RS, Parton WJ, Polley HW, Shaw MR 2004 Water relations in grassland and desert ecosystems exposed to elevated atmospheric CO_2_. Oecologia 140:11–25. 10.1007/s00442-004-1550-2 15156395

[PLV094C26] Nie M, Lu M, Bell J, Raut S, Pendall E 2013 Altered root traits due to elevated CO_2_: a meta-analysis. Global Ecology and Biogeography 22:1095–1105. 10.1111/geb.12062

[PLV094C27] Norby RJ, Luo Y 2004 Evaluating ecosystem responses to rising atmospheric CO_2_ and global warming in a multi-factor world. New Phytologist 162:281–293. 10.1111/j.1469-8137.2004.01047.x

[PLV094C28] Oram RN, Culvenor RA 1994 *Phalaris* improvement in Australia. New Zealand Journal of Agricultural Research 37:329–339. 10.1080/00288233.1994.9513071

[PLV094C29] Oram RN, Culvenor RA, Ridley AM 1993 Breeding the perennial pasture grass *Phalaris aquatica* for acid soils. In: Randall PJ, Delhaize E, Richards RA, Munns R, eds. Genetic aspects of plant mineral nutrition. The Netherlands: Springer, 17–22.

[PLV094C30] Oram RN, Ferreira V, Culvenor RA, Hopkins AA, Stewart A 2009 The first century of *Phalaris aquatica* L. cultivation and genetic improvement: a review. Crop and Pasture Science 60:1–15. 10.1071/CP08170

[PLV094C31] Poorter H, Van Berkel Y, Baxter R, Den Hertog J, Dijkstra P, Gifford RM, Griffin KL, Roumet C, Roy J, Wong SC 1997 The effect of elevated CO_2_ on the chemical composition and construction costs of leaves of 27 C_3_ species. Plant, Cell and Environment 20:472–482. 10.1046/j.1365-3040.1997.d01-84.x

[PLV094C32] Rachmilevitch S, Cousins AB, Bloom AJ 2004 Nitrate assimilation in plant shoots depends on photorespiration. Proceedings of the National Academy of Sciences of the USA 101:11506–11510. 10.1073/pnas.0404388101 15272076 PMC509230

[PLV094C33] Reich PB, Hobbie SE, Lee T, Ellsworth DS, West JB, Tilman D, Knops JMH, Naeem S, Trost J 2006 Nitrogen limitation constrains sustainability of ecosystem response to CO_2_. Nature 440:922–925. 10.1038/nature04486 16612381

[PLV094C34] Sardans J, Peñuelas J, Estiarte M, Prieto P 2008 Warming and drought alter C and N concentration, allocation and accumulation in a Mediterranean shrubland. Global Change Biology 14:2304–2316. 10.1111/j.1365-2486.2008.01656.x

[PLV094C35] Schenk HJ, Jackson RB 2002 The global biogeography of roots. Ecological Monographs 72:311–328. 10.1890/0012-9615(2002)072[0311:TGBOR]2.0.CO;2

[PLV094C36] Stitt M, Krapp A 1999 The interaction between elevated carbon dioxide and nitrogen nutrition: the physiological and molecular background. Plant, Cell and Environment 22:583–621. 10.1046/j.1365-3040.1999.00386.x

[PLV094C37] Stone L 2009 Environmental weed risk analysis: Phalaris aquatica. (ed. Culvenor RA). Perth, Australia: Future Farm Industries CRC.

[PLV094C38] Volder A, Edwards EJ, Evans JR, Robertson BC, Schortemeyer M, Gifford RM 2004 Does greater night-time, rather than constant, warming alter growth of managed pasture under ambient and elevated atmospheric CO_2_? New Phytologist 162:397–411. 10.1111/j.1469-8137.2004.01025.x

[PLV094C39] Volder A, Smart DR, Bloom AJ, Eissenstat DM 2005 Rapid decline in nitrate uptake and respiration with age in fine lateral roots of grape: implications for root efficiency and competitive effectiveness. New Phytologist 165:493–502. 10.1111/j.1469-8137.2004.01222.x 15720660

[PLV094C40] Volder A, Gifford RM, Evans JR 2007 Effects of elevated atmospheric CO_2_, cutting frequency, and differential day/night atmospheric warming on root growth and turnover of *Phalaris* swards. Global Change Biology 13:1040–1052. 10.1111/j.1365-2486.2007.01321.x

[PLV094C41] Vose RS, Easterling DR, Gleason B 2005 Maximum and minimum temperature trends for the globe: an update through 2004. Geophysical ResearchLetters; 10.1029/2005GL024379.

[PLV094C42] Wan S, Xia J, Liu W, Niu S 2009 Photosynthetic overcompensation under nocturnal warming enhances grassland carbon sequestration. Ecology 90:2700–2710. 10.1890/08-2026.1 19886480

[PLV094C43] Xu X, Sherry RA, Niu S, Li D, Luo Y 2013 Net primary productivity and rain-use efficiency as affected by warming, altered precipitation, and clipping in a mixed-grass prairie. Global Change Biology 19:2753–2764. 10.1111/gcb.12248 23649795

[PLV094C47] Zar JH 1984 Biostatistical analysis, 2nd edn New Jersey: Prentice Hall, 718.

[PLV094C44] Zhou X, Ge ZM, Kellomäki S, Wang KY, Peltola H, Martikainen P 2011 Effects of elevated CO_2_ and temperature on leaf characteristics, photosynthesis and carbon storage in aboveground biomass of a boreal bioenergy crop (*Phalaris arundinacea* L.) under varying water regimes. Global Change Biology Bioenergy 3:223–234. 10.1111/j.1757-1707.2010.01075.x

[PLV094C45] Ziska LH, Bunce JA 1995 Growth and photosynthetic response of three soybean cultivars to simultaneous increases in growth temperature and CO_2_. Physiologia Plantarum 94:575–584. 10.1111/j.1399-3054.1995.tb00970.x

[PLV094C46] Ziter C, MacDougall AS 2013 Nutrients and defoliation increase soil carbon inputs in grassland. Ecology 94:106–116. 10.1890/11-2070.1 23600245

