# Supplementary material for: Effects of elevated atmospheric CO2 concentrations, clipping regimen and differential day/night atmospheric warming on tissue nitrogen concentrations of a perennial pasture grass
Source: AoB Plants. 2015 Aug 13;7:plv094. doi: 10.1093/aobpla/plv094 (PMC4591745; doi:10.1093/aobpla/plv094)
Supplement: Additional Information [file supp_7_plv094_index.html]

Effects of elevated atmospheric CO2 concentrations, clipping regimen and differential day/night atmospheric warming on tissue nitrogen concentrations of a perennial pasture grass — Effects of elevated atmospheric CO2 concentrations, clipping regimen and differential day/night atmospheric warming on tissue nitrogen concentrations of a perennial pasture grass — Additional Information 

# Effects of elevated atmospheric CO2 concentrations, clipping regimen and differential day/night atmospheric warming on tissue nitrogen concentrations of a perennial pasture grass

## Additional Information

Additional Information

- Additional Information - Docx file
