# Supplementary material for: Effects of elevated atmospheric CO2 concentrations, clipping regimen and differential day/night atmospheric warming on tissue nitrogen concentrations of a perennial pasture grass
Source: AoB Plants. 2015 Aug 13;7:plv094. doi: 10.1093/aobpla/plv094 (PMC4591745; doi:10.1093/aobpla/plv094)
Supplement: Additional Information [file supp_plv094_plv094supp.docx]

**Supporting Information**

Table S1. Tissue N concentrations (mg N g^-1^) through time as affected by atmospheric CO_2_ concentration (403 µmol CO_2_ l^-1^ vs 759 µmol CO_2_ l^-1^), warming treatment, ambient (A), higher nighttime warming (HN) and continuous warming (CW), and clipping frequency (i, infrequent, f, frequent). Data presented are least-square-means based upon a full model including CO_2_ concentration, warming, and clipping frequency for each harvest period. Different letters indicate statistically significant differences at *P* <0.05 using Student’s T LSD test. $ = data ln transformed prior to analysis. *Post-hoc testing was not possible due to a missing treatment combination.

| Treat | 21 Nov – 19 Feb 2002 (Summer) | | | 20 Feb – 1 May 2002 (Fall) | | | 2 May – 25 Sep 2002 (Winter) | | |
| --- | --- | --- | --- | --- | --- | --- | --- | --- | --- |
| CO_2_-Warm-Clip | Leaf | Stem$ | Litter | Leaf | Stem | Litter | Leaf | Stem | Litter* |
| 403-A-i | 14.1 def | 5.18 b | 7.01 e | 17.9 def | 8.86 cd | 4.89 f | 18.2 bcde | 12.7 abc | ---- |
| 403-HN-i | 16.8 cd | 5.91 b | 8.09 abcde | 20.7 bcd | 10.6 abcd | 5.14 ef | 20.9 abcd | 10.6 cd | 6.07 |
| 403-C-i | 17.7 bc | 6.13 b | 8.81 abcd | 19.9 cde | 10.7 abcd | 4.73 f | 17.4 cde | 10.5 cd | 6.61 |
| 403-A-f | 20.3 ab | 11.8 a | 8.03 abcde | 23.7 ab | 10.7 abcd | 7.63 def | 25.3 a | 14.8 a | 6.48 |
| 403-HN-f | 20.3 ab | 10.8 a | 9.13 ab | 24.1 a | 12.3 ab | 14.3 bc | 24.8 a | 15.0 a | 7.81 |
| 403-C-f | 21.6 a | 9.76 a | 8.97 abc | 23.7 ab | 11.7 abc | 17.0 ab | 23.3 abc | 14.7 a | 9.70 |
|  |  |  |  |  |  |  |  |  |  |
| 759-A-i | 13.2 f | 5.73 b | 7.65 bcde | 17.0 efg | 9.01 cd | 5.57 ef | 17.9 cde | 11.4 bcd | 6.42 |
| 759-HN-i | 13.8 ef | 5.14 b | 7.42 de | 16.1 fg | 9.50 bcd | 5.10 ef | 16.2 de | 9.45 d | 4.82 |
| 759-C-i | 12.5 f | 4.43 b | 7.36 de | 14.3 g | 8.31 d | 5.48 ef | 14.0 e | 10.1 cd | 6.24 |
| 759-A-f | 18.3 bc | 11.3 a | 9.38 a | 22.3 abc | 8.41 d | 12.7 bcd | 26.6 a | 15.6 a | 7.99 |
| 759-HN-f | 17.3 c | 10.5 a | 9.56 a | 19.1 de | 10.3 abcd | 19.8 a | 23.7 abc | 14.4 ab | 5.07 |
| 759-C-f | 16.5 cde | 9.90 a | 7.55 cde | 20.5 cd | 13.0 a | 10.3 cde | 24.0 ab | 13.1 abc | 8.24 |
|  |  |  |  |  |  |  |  |  |  |
| CO_2_ | **<0.001** | 0.465 | 0.331 | **0.005** | *0.068* | 0.280 | 0.493 | 0.255 |  |
| Warm | 0.737 | 0.513 | 0.492 | 0.745 | 0.113 | 0.119 | 0.283 | 0.109 |  |
| CO_2_xW | 0.140 | 0.593 | *0.068* | 0.120 | 0.812 | 0.123 | 0.505 | 0.852 |  |
| Clip | **<0.001** | **<0.001** | **0.004** | **<0.001** | **0.025** | **<0.001** | **<0.001** | **<0.001** |  |
| CO_2_xClip | 0.694 | 0.517 | 0.322 | 0.586 | 0.912 | 0.692 | 0.217 | 0.712 |  |
| W x Clip | *0.096* | 0.795 | 0.149 | 0.140 | 0.354 | *0.058* | 0.695 | 0.639 |  |
| CO_2_ x W x Clip | 0.766 | 0.438 | 0.765 | 0.389 | 0.143 | **0.049** | 0.906 | 0.613 |  |
|  |  |  |  |  |  |  |  |  |  |
| Model R^2^ | 0.925 | 0.797 | 0.484 | 0.941 | 0.368 | 0.769 | 0.670 | 0.539 |  |
| Model *P* | <0.001 | <0.001 | <0.001 | <0.001 | <0.001 | <0.001 | <0.001 | <0.001 |  |

Table S1 continued. Tissue N concentrations (mg N g^-1^) through time

| Treat | 25 Sep – 11 Dec 2002 (Spring) | | | 12 Dec – 5 March 2003 (Summer) | | | 6 March – 20 May 2003 (Fall) | | |
| --- | --- | --- | --- | --- | --- | --- | --- | --- | --- |
| CO_2_-Warm-Clip | Leaf | Stem | Litter | Leaf | Stem | Litter$ | Leaf | Stem | Litter |
| 403-A-i | 18.3 d | 7.87 de | 6.53 de | 17.7 def | 8.17 bcd | 8.61 | 21.4 g | 11.7 | 5.27 f |
| 403-HN-i | 18.3 d | 8.46 d | 5.63 e | 21.1 bcd | 8.53 bcd | 13.6 | 25.2 def | 17.1 | 6.19 f |
| 403-C-i | 17.7 de | 8.03 d | 7.08 cde | 23.4 b | 11.0 ab | 17.7 | 24.2 efg | 12.1 | 8.07 def |
| 403-A-f | 23.9 bc | 11.1 bc | 8.43 bcde | 22.7 bc | 8.79 abcd | 9.01 | 31.8 ab | 17.2 | 22.8 a |
| 403-HN-f | 25.8 ab | 13.4 a | 21.6 a | 30.4 a | 11.4 a | 8.18 | 34.6 a | 20.6 | 15.6 abcd |
| 403-C-f | 26.1 a | 13.1 a | 13.9 b | 29.2 a | 10.6 abc | 10.8 | 32.0 ab | 13.7 | 16.6 abc |
|  |  |  |  |  |  |  |  |  |  |
| 759-A-i | 14.6 f | 6.24 e | 7.85 cde | 13.8 g | 8.23 bcd | 7.26 | 22.6 fg | 15.3 | 6.65 ef |
| 759-HN-i | 15.1 ef | 7.13 de | 4.70 e | 15.1 fg | 8.90 abcd | 12.1 | 20.9 g | 13.5 | 5.37 f |
| 759-C-i | 15.3 ef | 6.93 de | 5.61 e | 17.2 efg | 7.97 cd | 11.2 | 20.1 g | 12.7 | 4.98 f |
| 759-A-f | 21.7 c | 11.3 bc | 12.3 bc | 16.4 efg | 6.66 d | 6.39 | 30.8 abc | 15.7 | 10.4 cdef |
| 759-HN-f | 19.4 d | 10.1 c | 12.2 bcd | 19.1 cde | 7.06 d | 10.6 | 27.3 cde | 18.0 | 14.7 bcde |
| 759-C-f | 22.2 c | 12.2 ab | 24.9 a | 21.9 bc | 6.63 d | 7.49 | 29.0 bcd | 14.8 | 19.9 ab |
|  |  |  |  |  |  |  |  |  |  |
| CO_2_ | **0.009** | **0.008** | 0.656 | **<0.001** | **0.017** | 0.219 | **0.041** | 0.855 | 0.190 |
| Warm | 0.254 | **0.041** | **0.039** | **0.003** | 0.310 | 0.209 | 0.686 | 0.249 | 0.685 |
| CO_2_xW | 0.111 | *0.073* | **0.014** | 0.299 | 0.297 | 0.563 | **0.011** | 0.588 | 0.418 |
| Clip | **<0.001** | **<0.001** | **<0.001** | **<0.001** | 0.618 | 0.197 | **<0.001** | 0.119 | **<0.001** |
| CO_2_xClip | 0.319 | 0.955 | 0.333 | *0.057* | **0.030** | 0.813 | 0.420 | 0.733 | 0.417 |
| W x Clip | 0.378 | 0.396 | **0.006** | 0.292 | 0.567 | 0.687 | 0.789 | 0.872 | 0.821 |
| CO_2_ x W x Clip | 0.178 | 0.171 | **0.011** | 0.460 | 0.368 | 0.860 | 0.597 | 0.747 | **0.049** |
|  |  |  |  |  |  |  |  |  |  |
| Model R^2^ | 0.885 | 0.786 | 0.867 | 0.904 | 0.627 | -0.126 | 0.789 | 0.412 | 0.746 |
| Model *P* | <0.001 | <0.001 | <0.001 | <0.001 | <0.001 | <0.001 | <0.001 | <0.001 | <0.001 |

Table S2. Tissue carbon concentrations (g C g^-1^) through time as affected by atmospheric CO_2_ concentration (403 µmol CO_2_ l^-1^ vs 759 µmol CO_2_ l^-1^), warming treatment, ambient (A), higher nighttime warming (HN) and continuous warming (CW), and clipping frequency (I, infrequent, f, frequent). Data presented are least-square-means based upon a full model including CO_2_ concentration, warming, and clipping frequency for each harvest period. Different letters indicate statistically significant differences at *P* <0.05 using Student’s T LSD test. $ = data log transformed prior to analysis

| Treat | 21 Nov – 19 Feb 2002 (Summer) | | | 20 Feb – 1 May 2002 (Fall) | | | 2 May – 25 Sep 2002 (Winter) | | |
| --- | --- | --- | --- | --- | --- | --- | --- | --- | --- |
| CO_2_-Warm-Clip | Leaf | Stem$ | Litter | Leaf | Stem | Litter | Leaf | Stem | Litter |
| 403-A-i | 0.363 cd | 0.435 a | 0.358 bc | 0.410 bcde | 0.424 | 0.364 bcd | 0.428 | 0.431 ab | 0.365 a |
| 403-HN-i | 0.369 bcd | 0.431 a | 0.340 c | 0.414 bcde | 0.426 | 0.349 cde | 0.419 | 0.426 bc | 0.336 abcd |
| 403-C-i | 0.385 abc | 0.428 a | 0.378 b | 0.437 a | 0.418 | 0.377 abcd | 0.435 | 0.433 ab | 0.342 abcd |
| 403-A-f | 0.403 a | 0.370 b | 0.431 a | 0.412 bcde | 0.417 | 0.320 e | 0.423 | 0.433 ab | 0.337 bcd |
| 403-HN-f | 0.394 ab | 0.368 b | 0.427 a | 0.405 e | 0.416 | 0.371 abcd | 0.427 | 0.433 ab | 0.316 d |
| 403-C-f | 0.401 a | 0.360 b | 0.424 a | 0.416 bcde | 0.417 | 0.397 ab | 0.423 | 0.431 ab | 0.342 abcd |
|  |  |  |  |  |  |  |  |  |  |
| 759-A-i | 0.356 d | 0.423 a | 0.351 bc | 0.427 abd | 0.419 | 0.344 de | 0.430 | 0.420 a | 0.340 abcd |
| 759-HN-i | 0.381 abc | 0.433 a | 0.351 bc | 0.414 bcde | 0.415 | 0.366 bcd | 0.430 | 0.433 ab | 0.336 abcd |
| 759-C-i | 0.383 abc | 0.426 a | 0.357 bc | 0.424 abc | 0.418 | 0.373 abcd | 0.433 | 0.435 a | 0.366 ab |
| 759-A-f | 0.393 ab | 0.368 b | 0.418 a | 0.407 de | 0.422 | 0.344 de | 0.427 | 0.431 ab | 0.325 cd |
| 759-HN-f | 0.406 a | 0.360 b | 0.426 a | 0.406 e | 0.417 | 0.404 a | 0.427 | 0.435 a | 0.354 abc |
| 759-C-f | 0.397 a | 0.377 b | 0.436 a | 0.408 ce | 0.423 | 0.385 abc | 0.428 | 0.435 a | 0.321 cd |
|  |  |  |  |  |  |  |  |  |  |
| CO_2_ | 0.994 | 0.898 | 0.775 | 0.730 | 0.790 | 0.417 | 0.271 | 0.910 | 0.906 |
| Warm | 0.103 | 0.992 | 0.329 | **0.011** | 0.830 | **0.013** | 0.753 | 0.209 | 0.738 |
| CO_2_xW | 0.188 | 0.813 | 0.621 | *0.057* | 0.491 | 0.325 | 0.942 | *0.083* | 0.204 |
| Clip | **<0.001** | **<0.001** | **<0.001** | **0.010** | 0.657 | 0.267 | 0.199 | *0.068* | **0.014** |
| CO_2_xClip | 0.863 | 0.637 | 0.636 | 0.532 | 0.233 | 0.243 | 0.895 | 0.512 | 0.813 |
| W x Clip | 0.192 | 0.808 | 0.321 | 0.503 | 0.789 | **0.028** | 0.171 | 0.176 | 0.205 |
| CO_2_ x W x Clip | 0.996 | 0.682 | 0.168 | 0.336 | 0.920 | 0.382 | 0.291 | 0.248 | **0.022** |
|  |  |  |  |  |  |  |  |  |  |
| Model R^2^ | 0.602 | 0.844 | 0.935 | 0.103 | -0.324 | 0.697 | 0.752 | 0.608 | 0.770 |
| P | <0.001 | <0.001 | <0.001 | <0.001 | <0.001 | <0.001 | <0.001 | <0.001 | <0.001 |

.

Table S2. continued. Tissue Carbon concentrations through time.

| Treat | 25 Sep – 11 Dec 2002 (Spring) | | | 12 Dec – 5 March 2003 (Summer) | | | 6 March – 20 May 2003 (Fall) | | |
| --- | --- | --- | --- | --- | --- | --- | --- | --- | --- |
| CO_2_-Warm-Clip | Leaf$ | Stem | Litter | Leaf | Stem | Litter | Leaf | Stem | Litter |
| 403-A-i | 0.413 | 0.449 a | 0.370 cd | 0.423 abcd | 0.444 a | 0.389 abc | 0.420 ab | 0.421 a | 0.372 ab |
| 403-HN-i | 0.418 | 0.449 a | 0.358 cd | 0.418 bcde | 0.450 a | 0.405 abc | 0.425 ab | 0.415 a | 0.387 a |
| 403-C-i | 0.414 | 0.440 abc | 0.367 cd | 0.436 a | 0.451 a | 0.424 a | 0.423 ab | 0.411 ab | 0.388 a |
| 403-A-f | 0.414 | 0.434 abc | 0.376 bcd | 0.395 fg | 0.441 ab | 0.377 bc | 0.426 ab | 0.416 a | 0.413 a |
| 403-HN-f | 0.411 | 0.438 abc | 0.420 ab | 0.426 abc | 0.444 a | 0.370 bc | 0.433 a | 0.420 a | 0.367 ab |
| 403-C-f | 0.418 | 0.432 c | 0.364 cd | 0.416 bcde | 0.438 ab | 0.412 ab | 0.422 ab | 0.417 a | 0.382 a |
|  |  |  |  |  |  |  |  |  |  |
| 759-A-i | 0.396 | 0.444 abc | 0.382 bc | 0.402 efg | 0.440 ab | 0.367 c | 0.432 a | 0.419 a | 0.370 ab |
| 759-HN-i | 0.413 | 0.447 ab | 0.361 cd | 0.413 cde | 0.441 ab | 0.385 abc | 0.423 ab | 0.413 ab | 0.378 ab |
| 759-C-i | 0.413 | 0.437 abc | 0.367 cd | 0.433 ab | 0.447 a | 0.395 abc | 0.410 b | 0.409 ab | 0.371 ab |
| 759-A-f | 0.414 | 0.432 c | 0.371 bcd | 0.389 g | 0.420 cd | 0.369 c | 0.423 ab | 0.427 a | 0.323 b |
| 759-HN-f | 0.413 | 0.439 abc | 0.334 d | 0.408 def | 0.425 bcd | 0.380 bc | 0.422 ab | 0.415 a | 0.383 a |
| 759-C-f | 0.406 | 0.433 bc | 0.444 a | 0.415 cde | 0.438 ab | 0.365 c | 0.424 ab | 0.381 b | 0.397 a |
|  |  |  |  |  |  |  |  |  |  |
| CO_2_ | 0.351 | 0.455 | 0.946 | 0.108 | 0.272 | *0.070* | 0.658 | 0.272 | 0.254 |
| Warm | 0.364 | *0.063* | 0.360 | **0.004** | **0.019** | 0.208 | 0.560 | 0.155 | 0.554 |
| CO_2_xW | 0.510 | 0.841 | **0.019** | 0.471 | 0.168 | 0.426 | 0.584 | 0.336 | 0.225 |
| Clip | 0.613 | **0.020** | *0.084* | **<0.001** | **<0.001** | *0.068* | 0.479 | 0.773 | 0.947 |
| CO_2_xClip | 0.519 | 0.555 | 0.639 | 0.845 | 0.737 | 0.573 | 0.674 | 0.543 | 0.670 |
| W x Clip | 0.281 | 0.717 | 0.283 | **0.012** | 0.165 | 0.612 | 0.640 | 0.628 | 0.840 |
| CO_2_ x W x Clip | 0.233 | 0.990 | **0.013** | 0.157 | 0.470 | 0.435 | 0.199 | 0.360 | 0.135 |
|  |  |  |  |  |  |  |  |  |  |
| Model R^2^ | 0.363 | -0.642 | 0.704 | 0.877 | 0.602 | 0.637 | 0.603 | 0.113 | 0.207 |
| Model *P* | <0.001 | <0.001 | <0.001 | <0.001 | <0.001 | <0.001 | <0.001 | <0.001 | <0.001 |

Table S3 Tissue C:N ratios through time as affected by atmospheric CO_2_ concentration (403 µmol CO_2_ l^-1^ vs 759 µmol CO_2_ l^-1^), warming treatment, ambient (A), higher nighttime warming (HN) and continuous warming (CW), and clipping frequency (I, infrequent, f, frequent). Data presented are least-square-means based upon a full model including CO2 concentration, warming, and clipping frequency for each harvest period. Different letters indicate statistically significant differences at *P* <0.05 using Student’s T LSD test. $ = data ln transformed prior to analysis. * Post-hoc testing was not possible due to a missing treatment combination.

| Treat | 21 Nov – 19 Feb 2002 (Summer) | | | 20 Feb – 1 May 2002 (Fall) | | | 2 May – 25 Sep 2002 (Winter) | | |
| --- | --- | --- | --- | --- | --- | --- | --- | --- | --- |
| CO_2_-Warm-Clip | Leaf | Stem | Litter | Leaf | Stem | Litter | Leaf | Stem | Litter* |
| 403-A-i | 26.0 bcd | 84.2 ab | 51.3 abc | 23.1 bcd | 48.3 abc | 74.5 ab | 23.8 bc | 33.9 cd | ---- |
| 403-HN-i | 22.0 defgh | 73.8 b | 42.0 d | 20.2 def | 40.9 abcd | 70.5 ab | 20.5 bcde | 40.6 abc | 56.2 |
| 403-C-i | 22.0 defg | 69.9 b | 43.6 cd | 22.0 cd | 39.8 abcd | 79.8 a | 26.4 ab | 42.5 ab | 54.1 |
| 403-A-f | 20.0 fgh | 31.7 c | 54.1 ab | 17.4 fg | 39.3 abcd | 44.1 c | 16.9 e | 29.8 d | 53.2 |
| 403-HN-f | 19.5 gh | 34.1 c | 47.1 bcd | 16.8 g | 34.1 cd | 30.7 cd | 17.5 de | 29.1 d | 44.8 |
| 403-C-f | 18.6 h | 37.6 c | 47.3 bcd | 17.6 fg | 36.0 bcd | 25.3 d | 18.7 cde | 29.5 d | 37.0 |
|  |  |  |  |  |  |  |  |  |  |
| 759-A-i | 26.8 abc | 75.7 b | 46.0 bcd | 25.2 bc | 47.4 abcd | 61.9 b | 24.0 bcd | 36.8 bcd | 53.2 |
| 759-HN-i | 28.0 ab | 85.8 ab | 47.8 bcd | 25.9 b | 48.1 abcd | 71.8 ab | 26.7 ab | 46.2 a | 71.1 |
| 759-C-i | 30.8 a | 96.3 a | 49.7 abcd | 30.1 a | 52.5 a | 68.1 ab | 31.1 a | 43.3 ab | 58.6 |
| 759-A-f | 21.4 efgh | 33.1 c | 44.6 cd | 18.3 efg | 50.2 ab | 30.0 cd | 16.6 e | 28.6 d | 40.7 |
| 759-HN-f | 23.5 cdef | 34.4 c | 44.9 cd | 21.4 cde | 41.2 abcd | 21.5 d | 18.4 cde | 30.9 d | 53.4 |
| 759-C-f | 24.1 bcde | 38.3 c | 57.7 a | 20.2 def | 33.1 d | 37.2 cd | 18.7 cde | 33.7 cd | 41.4 |
|  |  |  |  |  |  |  |  |  |  |
| CO_2_ | **<0.001** | 0.286 | 0.596 | **0.005** | *0.077* | **0.025** | 0.359 | *0.074* |  |
| Warm | 0.874 | 0.402 | 0.227 | 0.366 | 0.245 | 0.615 | *0.074* | **0.042** |  |
| CO_2_xW | *0.098* | *0.081* | **0.031** | 0.205 | 0.936 | 0.361 | 0.448 | 0.712 |  |
| Clip | **<0.001** | **<0.001** | 0.127 | **<0.001** | **0.037** | **<0.001** | **<0.001** | **<0.001** |  |
| CO_2_xClip | 0.201 | 0.219 | 0.412 | **0.045** | 0.832 | 0.560 | 0.175 | 0.677 |  |
| W x Clip | 0.344 | 0.957 | 0.347 | *0.097* | 0.544 | 0.217 | 0.356 | 0.260 |  |
| CO_2_ x W x Clip | 0.421 | 0.158 | 0.290 | 0.263 | 0.226 | 0.127 | 0.675 | 0.622 |  |
|  |  |  |  |  |  |  |  |  |  |
| Model R^2^ | 0.862 | 0.861 | 0.653 | 0.912 | 0.287 | 0.820 | 0.728 | 0.435 |  |
| Model *P* | <0.001 | <0.001 | <0.001 | <0.001 | <0.001 | <0.001 | <0.001 | <0.001 |  |

S3 continued

| Treat | 25 Sep – 11 Dec 2002 (Spring) | | | 12 Dec – 5 March 2003 (Summer) | | | 6 March – 20 May 2003 (Fall) | | |
| --- | --- | --- | --- | --- | --- | --- | --- | --- | --- |
| CO_2_-Warm-Clip | Leaf | Stem | Litter | Leaf | Stem | Litter | Leaf | Stem | Litter |
| 403-A-i | 22.6 b | 57.2 bc | 56.9 ab | 24.1 bcd | 54.5 abcd | 45.1 | 19.6 ab | 36.2 | 72.6 a |
| 403-HN-i | 22.9 b | 53.4 cd | 64.1 ab | 20.4 def | 53.9 abcd | 29.8 | 17.3 bcd | 25.2 | 65.4 a |
| 403-C-i | 23.4 b | 55.2 bc | 52.7 bcd | 19.0 ef | 44.6 cd | 23.9 | 17.8 abc | 35.2 | 54.6 ab |
| 403-A-f | 17.4 d | 39.2 ef | 61.6 ab | 17.4 fg | 47.3 bcd | 41.7 | 13.4 ef | 24.5 | 18.5 c |
| 403-HN-f | 15.9 d | 33.0 f | 14.0 e | 14.0 g | 39.2 d | 43.7 | 12.5 f | 20.4 | 26.1 c |
| 403-C-f | 16.1 d | 33.1 f | 31.7 cde | 14.3 g | 41.4 d | 38.1 | 13.2 ef | 58.7 | 24.9 c |
|  |  |  |  |  |  |  |  |  |  |
| 759-A-i | 27.2 a | 74.5 a | 48.9 bc | 29.1 a | 57.0 abcd | 50.3 | 19.2 ab | 27.8 | 56.2 ab |
| 759-HN-i | 27.4 a | 63.3 b | 77.7 a | 27.4 ab | 49.7 bcd | 31.7 | 20.4 a | 31.4 | 70.4 a |
| 759-C-i | 27.2 a | 66.6 b | 66.1 ab | 25.5 abc | 57.0 abcd | 35.2 | 20.5 a | 33.3 | 83.0 a |
| 759-A-f | 19.1 cd | 38.2 ef | 39.4 bcde | 24.0 bcd | 63.4 ab | 57.7 | 13.7 ef | 40.1 | 31.3 bc |
| 759-HN-f | 21.8 bc | 44.4 de | 26.6 de | 21.6 cde | 60.8 abc | 36.0 | 15.5 cde | 23.1 | 30.7 bc |
| 759-C-f | 18.3 d | 35.5 f | 22.1 cde | 18.9 ef | 70.5 a | 48.6 | 14.7 def | 45.8 | 20.2 c |
|  |  |  |  |  |  |  |  |  |  |
| CO_2_ | **0.012** | **0.020** | 0.997 | **<0.001** | **0.045** | 0.338 | *0.081* | 0.980 | 0.460 |
| Warm | *0.061* | **0.013** | 0.255 | **0.008** | 0.612 | 0.244 | 0.960 | 0.281 | 0.769 |
| CO_2_xW | **0.005** | 0.200 | *0.065* | 0.643 | 0.380 | 0.654 | *0.057* | 0.831 | 0.997 |
| Clip | **<0.001** | **<0.001** | **<0.001** | **<0.001** | 0.761 | 0.276 | **<0.001** | 0.664 | **<0.001** |
| CO_2_xClip | 0.480 | 0.116 | 0.206 | 0.945 | **0.011** | 0.880 | 0.876 | 0.859 | 0.773 |
| W x Clip | 0.562 | 0.363 | **0.006** | 0.977 | 0.638 | 0.746 | 0.742 | 0.503 | 0.807 |
| CO_2_ x W x Clip | 0.499 | 0.238 | 0.664 | 0.693 | 0.714 | 0.879 | 0.807 | 0.711 | 0.158 |
|  |  |  |  |  |  |  |  |  |  |
| Model R^2^ | 0.302 | 0.702 | 0.807 | 0.838 | 0.695 | -0.243 | 0.785 | 0.203 | 0.726 |
| Model *P* | <0.001 | <0.001 | <0.001 | <0.001 | <0.001 | <0.001 | <0.001 | <0.001 | <0.001 |
